# Magnetic resonance imaging tracking and assessing repair function of the bone marrow mesenchymal stem cells transplantation in a rat model of spinal cord injury

**DOI:** 10.18632/oncotarget.19775

**Published:** 2017-08-01

**Authors:** Hongwu Zhang, Liqin Wang, Shihong Wen, Qingfeng Xiang, Xianhong Xiang, Caixia Xu, Yong Wan, Jingnan Wang, Bin Li, Yiqian Wan, Zhiyun Yang, David Y .B. Deng

**Affiliations:** ^1^ Research Center of Translational Medicine, The First Affiliated Hospital, Sun Yat-Sen University, Guangzhou 510080, China; ^2^ Department of Anatomy, Guangdong Provincial Key Laboratory of Construction and Detection in Tissue Engineering, Southern Medical University, Guangzhou 510515, China; ^3^ Department of Interventional Radiology, The First Affiliated Hospital, Sun Yat-Sen University, Guangzhou 510080, China; ^4^ The First People's Hospital of Foshan, Foshan 528000, China; ^5^ Department of Spine Surgery, The First Affiliated Hospital, Sun Yat-Sen University, Guangzhou 510080, China; ^6^ School of Chemistry and Chemical Engineering, Sun Yat-Sen University, Guangzhou 510275, China; ^7^ Guangdong Provincial Key Laboratory of Orthopedics and Traumatology, The First Affiliated Hospital, Sun Yat-Sen University, Guangzhou 510080, China

**Keywords:** Gd-DTPA-FA, endothelial lipase, bone marrow mesenchymal stem cells, magnetic resonance imaging, spinal cord injury

## Abstract

The transplantation of bone marrow mesenchymal stem cells (BMSCs) to repair spinal cord injury (SCI) has become a promising therapy. However, there is still a lack of visual evidence directly implicating the transplanted cells as the source of the improvement of spinal cord function. In this study, BMSCs were labeled with NF-200 promoter and lipase-activated gadolinium-containing nanoparticles (Gd-DTPA-FA). Double labeled BMSCs were implanted into spinal cord transaction injury in rat models *in situ*, the function recovery was evaluated on 1st, 7th, 14th, 28 th days by MRI, Diffusion Tensor Imaing, CT imaging and post-processing, and histological observations. BBB scores were used for assessing function recovery. After transplantation of BMSCs, the hypersignal emerged in spinal cord in T1WI starting at day 7 that was focused at the injection site, which then increased and extended until day 14. Subsequently, the increased signal intensity area rapidly spread from the injection site to entire injured segment lasting four weeks. The diffusion tensor tractography and histological analysis both showed the nerve fibre from dividing to connecting partly. Immunofluorescence showed higher expression of NF-200 in Repaired group than Injury group. Electron microscopy showed detachment and loose of myelin lamellar getting better in Repaired group compared with the Injury group. BBB scores in Repaired group were significantly higher than those of injury animals. Our study suggests that the migration and distribution of Gd-DTPA-FA labeled BMSCs can be tracked using MRI. Transplantation of BMSCs represents a promising potential strategy for the repair of SCI.

## INTRODUCTION

Spinal cord injury (SCI) is a common trauma of the central nervous system, and morbidity associated with SCI is high [1]. In recent years, the transplantation of exogenous stem cells to repair the spinal cord has become the focus of much research. Stem cells have the potential for multi-directional differentiation and can differentiate into neuron-like cells at the site of the SCI after transplantation.

Bone marrow mesenchymal stem cells (BMSCs) are non-hematopoietic stem cells found in high abundance in the reticular stroma. Characteristics like its abundance, its high capacity for self-renewal, pluripotency, weak immunogenicity, and ease of transfection make BMSCs as an ideal source of stem cells for transplantation [2]. While many groups have shown that the transplantation of BMSCs can at least partially restore spinal function, the mechanism behind this recovery is currently unclear. On one hand, Wu et al. [3] have proposed that the mechanism of BMSC-promoted recovery of spinal cord function may be closely related to its promotion of the differentiation of endogenous NSCs. On the other hand, Hofstetter et al. [4] have suggested that BMSC-promoted recovery may not be due to BMSCs replacing the damaged neurons but rather occurs due to axonal regeneration of nascient spinal cord neurons based on the distribution characteristics of BMSCs. Although these studies have proposed different explanations for how BMSCs promote the repair of SCI, it is indisputable that each group has observed functional recovery of SCI after the transplantation of BMSCs. In previous studies, we induced BMSCs to differentiate into neuron-like cells *in vitro*, and transplanted these neuron-like cells into a rhesus SCI model. We observed that BMSCs not only survived but also expressed neuronal markers led to improved motor function significantly [5]. The recovery was found to be more pronounced with the addition of neurotrophic factor [6]. Clinical trials have confirmed the safety of BMSC transplantation [7–9], and these results serve as a reference for the clinical transplantation of BMSCs and the development of future treatment strategies. The ability of BMSCs to differentiate into nerve cells has important research implications and promising applications in the treatment of neurological disorders.

Many questions remain unsolved regarding the transplantation of BMSCs to repair spinal cord injuries. For instance, researchers do not know whether the transplanted cells or other autologous cells play the leading role after stem cell transplantation into the body. Furthermore, it is unclear precisely how transplanted stem cells survive, differentiate, and migrate *in vivo*. In order to track the migration, differentiation and distribution of transplanted BMSCs *in vivo*, most groups have relied upon conventional tests like labeling transplanted cells with Green fluorescent protein or 5-bromodeoxyuridine. However, while these methods allow for the detection of transplanted cells at select time points using immunohistochemistry and immunofluorescence methods [10], they do not allow for real-time *in vivo* observation of migration, distribution or differentiation of transplanted cells.

The use of Magnetic resonance imaging (MRI) molecular morphology imaging to track transplanted stem cells is well-established, and magnetic tags are commonly used to visualize and track stem cells after transplantation into the body. Gadolinium (Gd) labels were used as early as 2000, when Louie [11] used polymer particles within gadolinium-dimethylene pentaacetic acid (Gd-DTPA) to label transplanted cells. When used for MRI, the Gd label shows a high T1WI that is unaffected by air, and the FDA approved the use of Gd-DTPA as an MRI contrast agent for clinical diagnosis in 1987. While Gd labels have certain advantages in terms of tracking stem cells, a major weakness of the Gd label is its lack of specificity [12–14].

Previously, we synthesized a new nanomaterial, Gd-DTPA-FA, through the addition of two straight saturated hydrocarbon chains to the Gd-DTPA molecule. Using the lipid “shell” formed by two long aliphatic chains to coat Gd-DTPA, we increased the hydrophobicity of the particle and shielded its signal positivity in MRI T1WI. After treatment with lipase to sever the ester bond combining the aliphatic chains, the hydrophilic properties of the molecule were restored to increase contrast activity and demonstrate the effectiveness of Gd-DTPA-FA as a lipase activated magnetic resonance contrast agent [15]. Additionally, we constructed endothelial lipase lentiviral vectors carrying a promoter for neurofilament-200 (NF-200). NF-200 is expressed in the cytoplasm and axon of nerve cells and can be used to track the neuron-like cells that are derived from the stem cells transplanted to repair SCI [16]. Using the lentiviral vector and the nanoparticle Gd-DTPA-FA, we were able to ensure that only neuron-like cells expressing NF-200 will exhibit a positive signal change in MRI T1WI *in vivo*.

In the present study, we describe an ideal method for dynamic *in vivo* monitoring of a target gene in real-time by using MRI. Additionally, we synthesized lipase-activated gadolinium-containing nanoparticles—Gd-DTPA-FA and constructed a lentiviral vector encoding endothelial lipase to track the expression of NF-200 in transplanted BMSCs. As applied to stem cells transplanted in a SCI model, these nanoparticles and lentiviral tools offer a novel method to monitor transplanted cells and provide further support for the role of BMSCs in promoting the recovery of nerve function after injury.

## RESULTS

### Surface antigen expression of BMSCs and lentiviral infection

**Figure 1:**
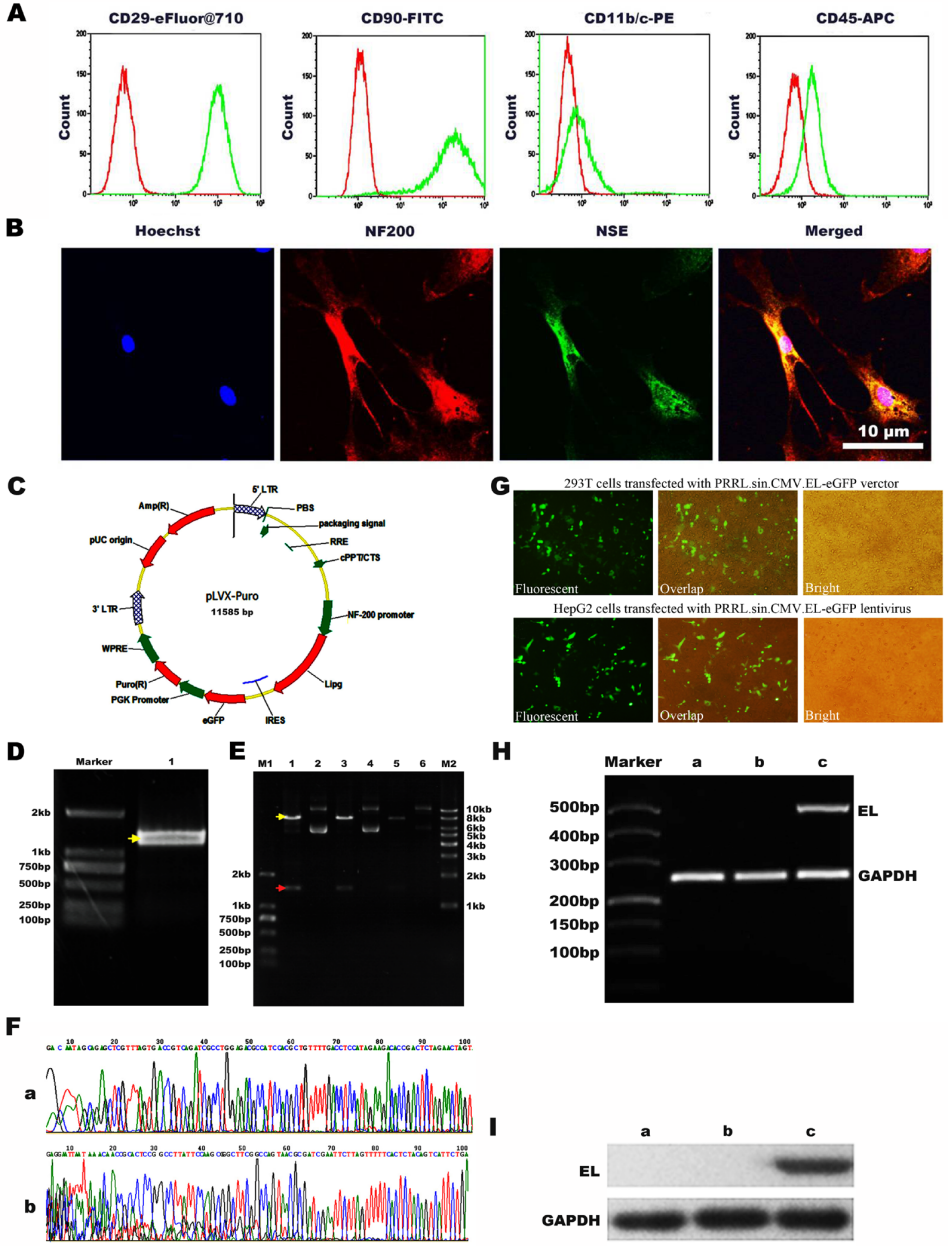
Immunophenotypic analysis of BMSCs and the construction of NF-200 promoter of lentivirus plasmid and identification (**A**) Flow cytometry showed negative expression of CD45 and CD11b/c while positive expression of CD29 and CD90. (**B**) Confocal microscopy images to confirm the neurogenic differentiation of BMSCs by the neurocyte-specific markers NSE and NF-200. Hoechst33342 a blue tint to the nucleus, NF-200 dyed red, and NSE dyed green. (**C**) Scheme of plasmid. (**D**) Agarose gel electrophoresis of EL (endothelial lipase) DNA. (**E**) PRRL. sin. CMV. EL-eGFP digested with DraI and NotI enzymes1, 3, 5: DNA extracted from three stochastic monoclonal colonies of PRRL. sin. CMV. EL-eGFP; 2, 4, 6: Double digestion results of 1, 3, 5 with SpeI and EcoRI. (**F**) Sequencing of the recombined PRRL. sin. CMV. EL-eGFP verctor. a: The sequenceing after Pcmv; b: The sequenceing before IRES. (**G**) Production of recombined lentivirus and infection with HepG2 cells. a: Fluorescent image of 293T cells transfected with PRRL. sin. CMV. EL-eGFP verctor; b: Overlap image of 293T cells transfected with PRRL. sin. CMV. EL-eGFP verctor; c: Bright image of 293T cells transfected with PRRL. sin. CMV. EL-eGFP verctor; d: Fluorescent image of HepG2 cells transfected with PRRL. sin. CMV. EL-eGFP lentivirus; e: Overlap image of HepG2 cells infected with PRRL. sin. CMV. EL-eGFP lentivirus; f: Bright image of HepG2 cells transfected with PRRL. sin. CMV. EL-eGFP lentivirus. (**H**) Expressing of EL mRNA in HepG2 cells. a: untransfected HepG2 cells; b: transfected HepG2 cells with PRRL. sin. CMV. eGFP lentivirus; c: transfected HepG2 cells with PRRL. sin. CMV. EL-eGFP lentivirus; EL: endothelial lipase. (**I**) Expressing of EL protein in HepG2 cells. a. untransfected HepG2 cells; b. transfected HepG2 cells with PRRL. sin. CMV. eGFP lentivirus; c. infected HepG2 cells with PRRL. sin. CMV. EL-eGFP lentivirus.

Flow cytometry showed that BMSCs are highly homogeneous in phenotype. All cells were CD29+, CD90+, CD11b/c– and CD45- (Figure 1A). After the BMSCs differentiated into neuron-like cells, the expression of NF-200 and NSE was observed by immunofluorescence microscopy (Figure 1B). And the rate of the neurogenic differentiation of BMSCs is about 70%. For cell transduction and virus vectors were prepared as previously described [17]. The recombined lentivirus vector with NF-200 promoter was produced and the infected rate was examed. The HepG2 cells were plated into 24-well poly-L-lysine coated plates in DMEM for 2 days. Virus-contained solution was then added directly to culture DMEM media and incubated at 37°C for 48 h. GFP expression was visualized by fluorescence microscopy after 48 h and the numbers of total cells and GFP positive cells were assessed to determine transduction efficiency and viability. The transduction efficiency was then calculated using the ratio of the number of GFP-positive cells to the total cell number (Figure 1C–1I).

### CT scan results

As the CT images shown for all groups 1 day after injection, the hemorrhages resolved spontaneously in all cases. The presence of hemorrhage abrogated the distinction of injured spinal cord from surrounding tissue in the region, as visualized by CT imaging. However, in post-processing color images, the boundary between the injuried spinal cord and adjacent tissue was distinguishable. The green hematoma was noted to be distinct from blue spinal cord in Injury group. Blue thick funiculose tissue was noted to be connected to the normal blue spinal cord in Repaired group (Supplementary Figure 1).

### MR imaging tracing the differentiation, migration, and distribution of BMSCs

**Figure 2:**
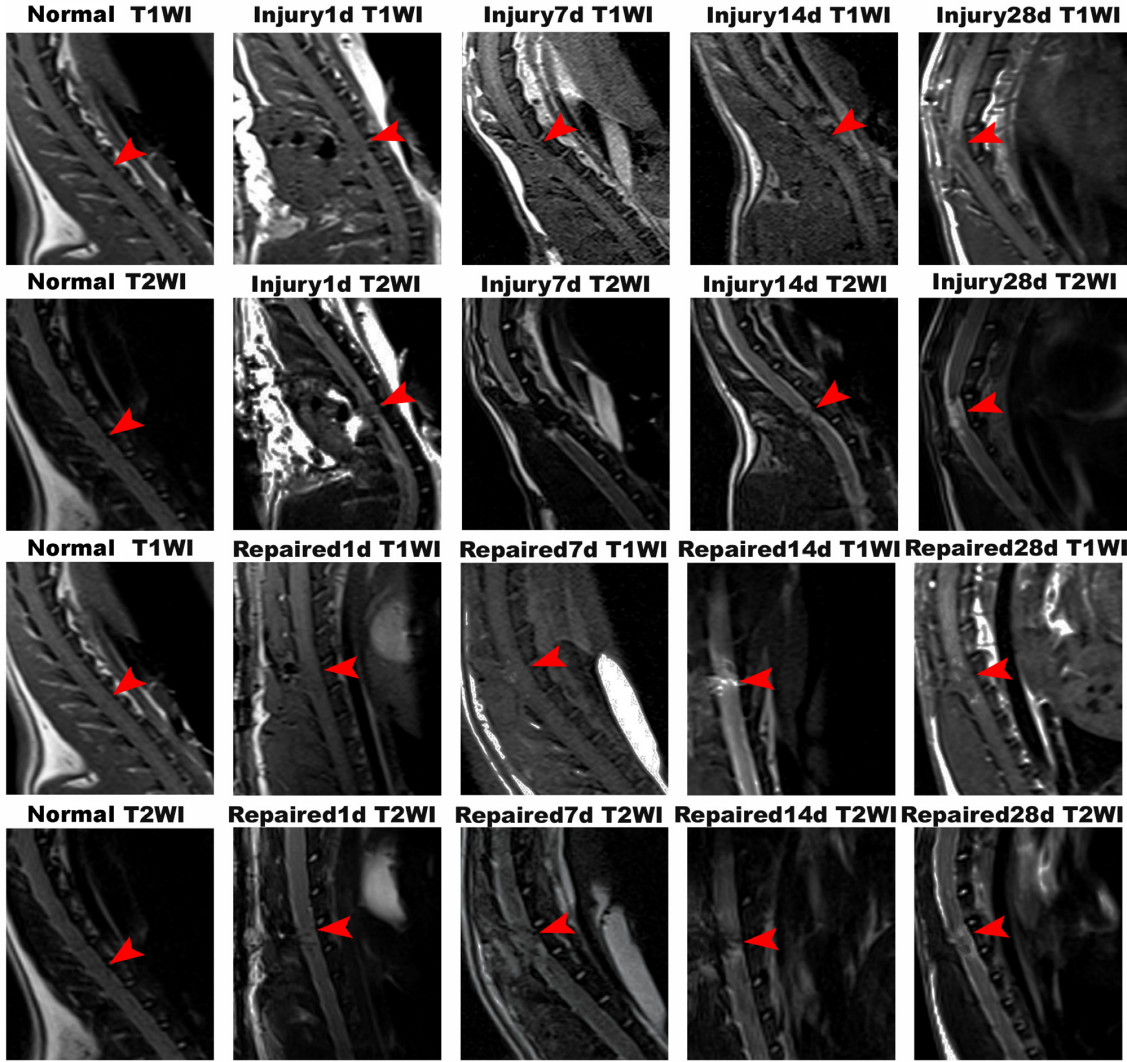
MRI imaging of Normal group, Injury group and Repaired group in the rat SCI model at 1 d, 7 d, 14 d and 28 d In Normal group, the rat spinal cord was continuous and uninterrupted. In Injury group, there was no observable increase in signal intensity of the spinal cord in T1WI from 1st day to 28th day. In contrast, hypersignal emerged in spinal cords of the Repaired group of rats in T1WI beginning on the 7th day, which was further observed to be increased and extended until the 14th day statu-post injection. On day 28, the area of hypersignal in spinal cord was larger than before. Arrows indicated the segmental SCI.

As shown, the spinal cord signal intensity in T1WI and T2WI was uniform in the normal group. In the Injury group, there was no observable increase in signal intensity of the spinal cord in T1WI from 1st day to 28th day (except within the cavity formation). In contrast, hypersignal emerged in spinal cords of the Repaired group of rats in T1WI beginning on the 7th day, which was focused in injection site. This hypersignal was observed to increase and extend until the 14th day statu-post injection. On day 28, the area of hypersignal in spinal cord was larger than before (Figure 2; Supplementary Figure 2).

### BMSCs transplantation enhanced the structural and functional recovery after SCI

**Figure 3:**
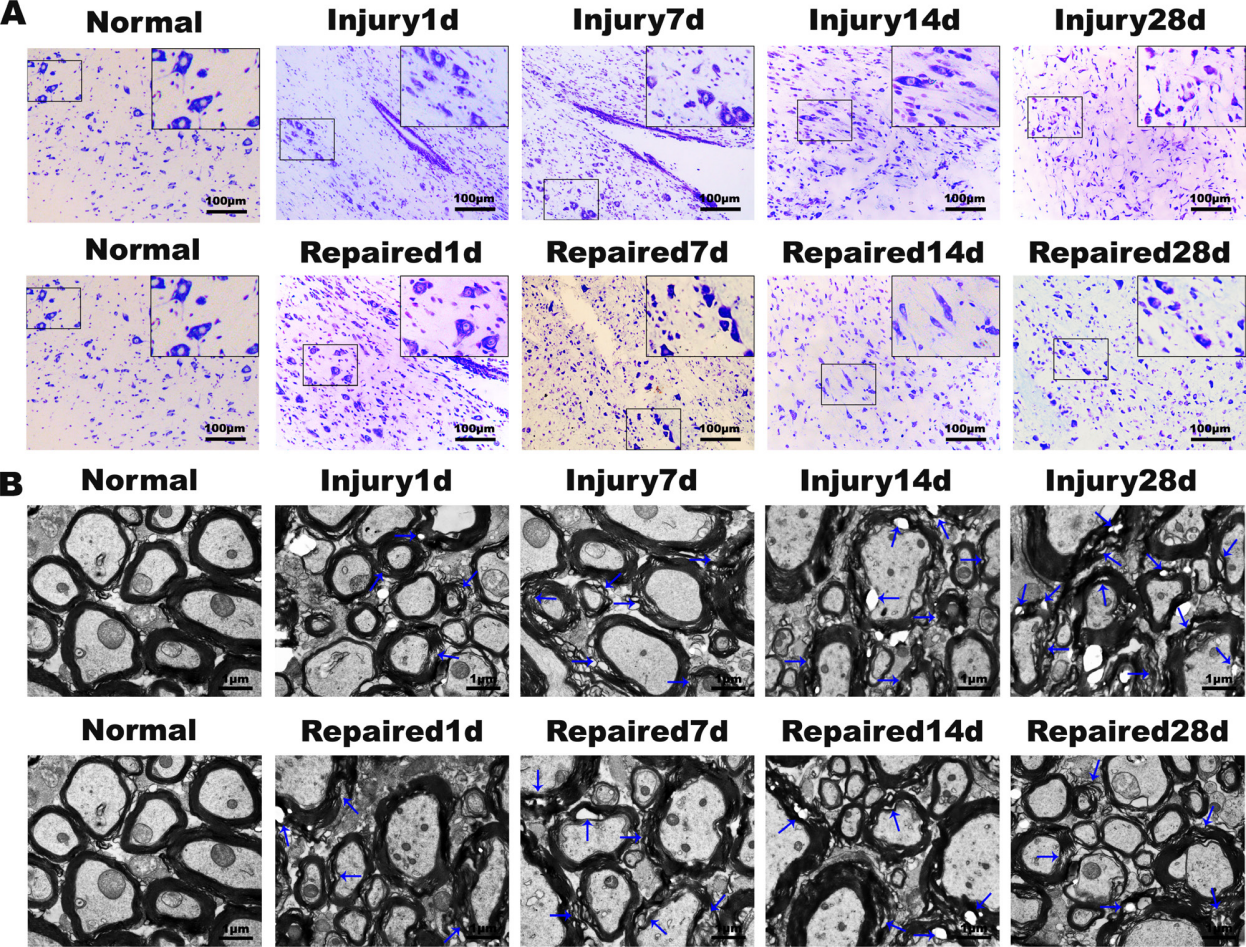
Nissl's staining and electron microscopy of rat spinal cord tissue (**A**) Nissl's staining results showed that the numbers of neurons decreased gradually from the 1st day to 28th day in Injury group, and the damage degree increased gradually. In Repaired group, corresponding BMSCs transplantation could promote the survival of neurons, reduce the apoptosis of SCI (magnification: 100×; insert magnification: 400 ×). (**B**) The electron microscopy scans. The Injury group and Repaired group showed many demyelinating axons. In Injury group, the degree of pathological changes of ultrastructure over time decreased, and the ultrastructure of BMSCs transplantation group was obviously improved.

Nissl staining of longitudinal sections in the normal group demonstrated that the neuronal structure was normal and uniformly distributed. Within the Repaired group, a decrease in the number of neurons and abnormal structure was observed at day 1, as compared to Injury. The normal structure of the cord gradually returned from day 14 to 28, and the neurons were observed to achieve an even distribution after this time (Figure 3A). Scanning electron microscopy showed that the myelin of the spinal cord in the Normal group maintained structural integrity and was organized in a tight concentric ring. In the Repaired group, the ultrastructure of spinal cord demonstrated significant normalization between day 14 and 28, demonstrating a much smaller cavity within the myelin (Figure 3B).

**Figure 4:**
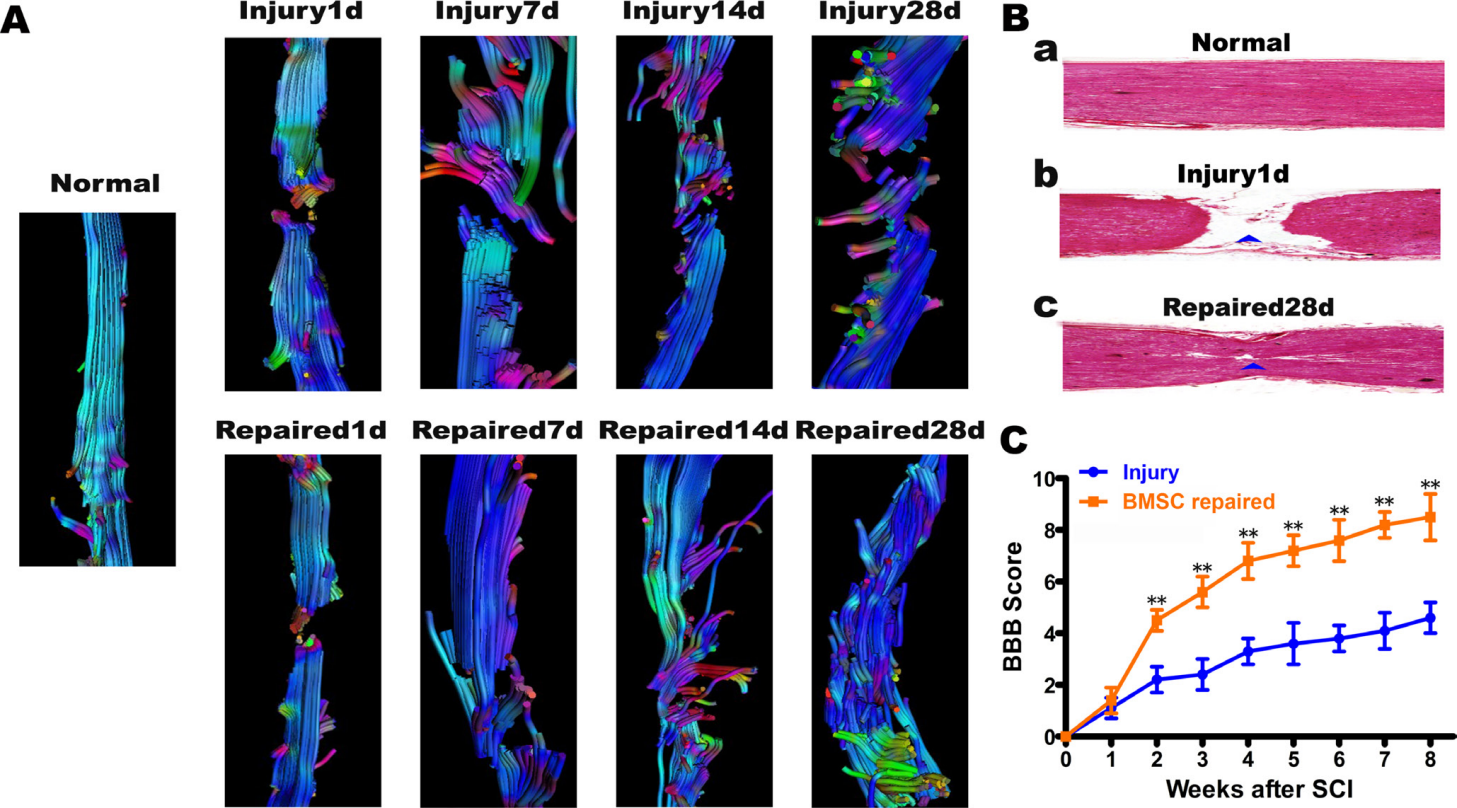
Diffusion tensor tractography (DTT) figures, H.E staining of rat spinal cord tissue and BBB locomotor function score (**A**) The DTT. In Injury group, the spinal cord fibers continuity were interrupted from the 1st day to 28th day, and end fibers arranged disorderly, colors were uneven; In Repaired group, the nerve fibers from the 1st day to 28th day, some fibers connected to the other end. The color was almost consistent, but the arrangement of neurofibra was still disorder. (**B**) a. In Normal group, visible normal spinal structure; b. In Injury 1d group, spinal cord was completely broken; c. In Repaired 28 d group, continuous spinal nerve fibers appeared in SCI site. (**C**) BBB locomotor function score. BBB scores increased gradually from 2～8 weeks in BMSCs repaired group. After transplant 8 weeks, BBB scores (score > 8)were significantly higher than Injury group(score < 4), **P < 0.01.

**Figure 5:**
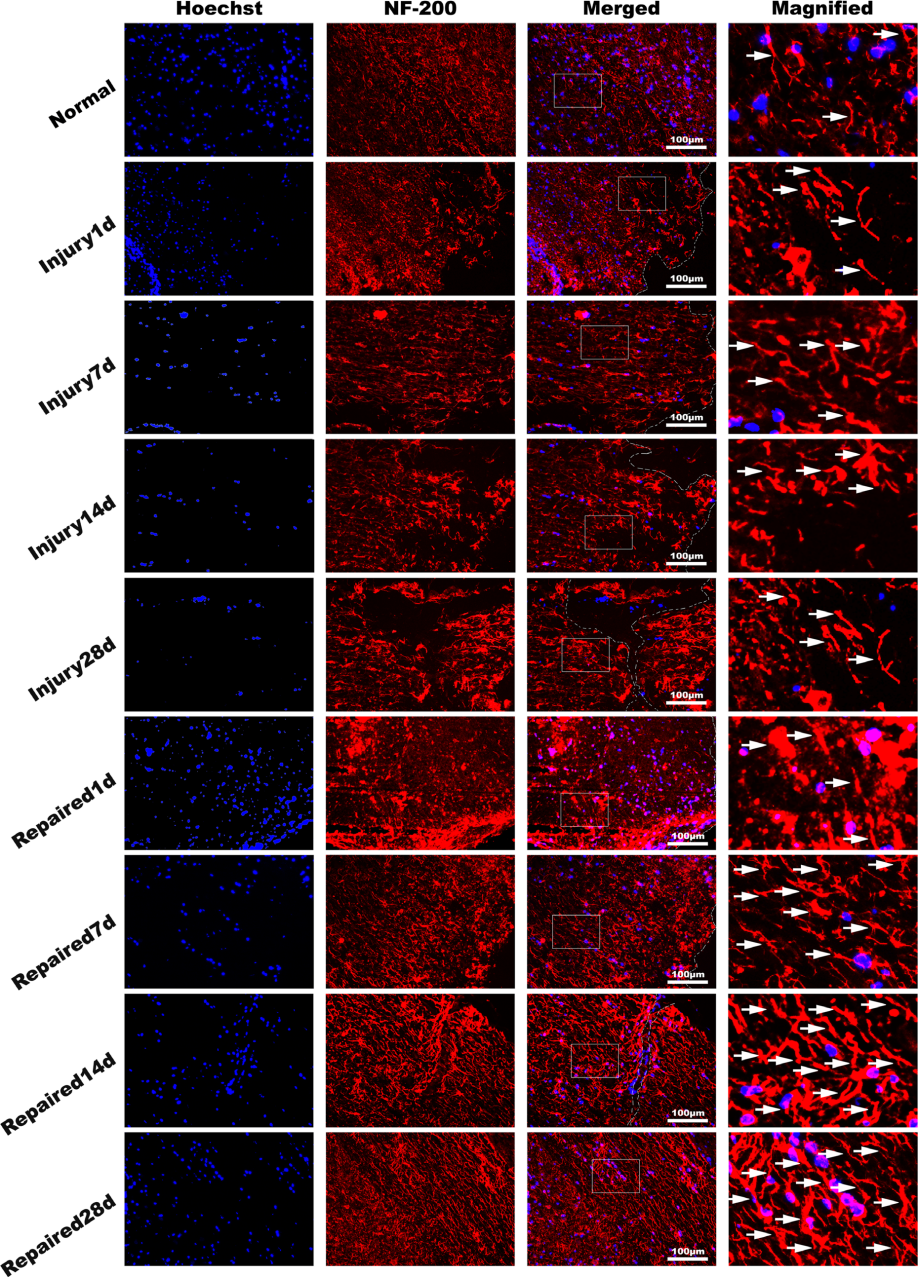
Immunofluorescence of spinal cord tissue NF-200 antibody (red), dyeing Hoechst33342 nucleus (blue). It showed that the pathological changes in Injury group decreased with time obviously in longitudinal section. The NF-200 positive cells expressing quantity increased significantly in Repaired group compared to Injury group (*n* = 5). Bar: 100 μm.

The 3D-T1W1, DTI sequence scan, and DTT post-processing were measured by Siemens Verio 3D neuro software. In the repaired group, DTT showed that a population of continuous nerve fibers that gradually increased from day 1 to day 28. At day 28, approximately half the diameter of the spinal cord fibers were connected but arranged in an irregular pattern (Figure 4A). H.E staining of rat spinal cord tissue in the Normal, Injury1d and Repaired 28d group demonstrated that continuous spinal nerve fibers appeared in SCI site increasingly (Figure 4B). BMSC transplantation was found to promote the functional recovery of SCI using objective measurements. In all injured animals, the hind legs were completely paralyzed after the rat spinal cord was transected completely. However, in the BMSC-transplanted repaired group, BBB scores increased gradually from 2～8 weeks. At 8 weeks status-post transection, BBB scores (score > 8) were significantly higher than Injury group (score < 4), **P < 0.01 (Figure 4C). Immunofluorescence staining of NF-200 revealed that BMSCs were induced into neuron-like cells. The expression of neurofilament protein NF-200 increased gradually in a disordered fashion within the Injury group. In the Repaired group, however, the expression of NF-200 normalized, and the neuron-like cell populations appeared to have a more regular arrangement than Injury (Figure 5).

### Differentiation, migration and distribution diagram of transplantated BMSCs

**Figure 6:**
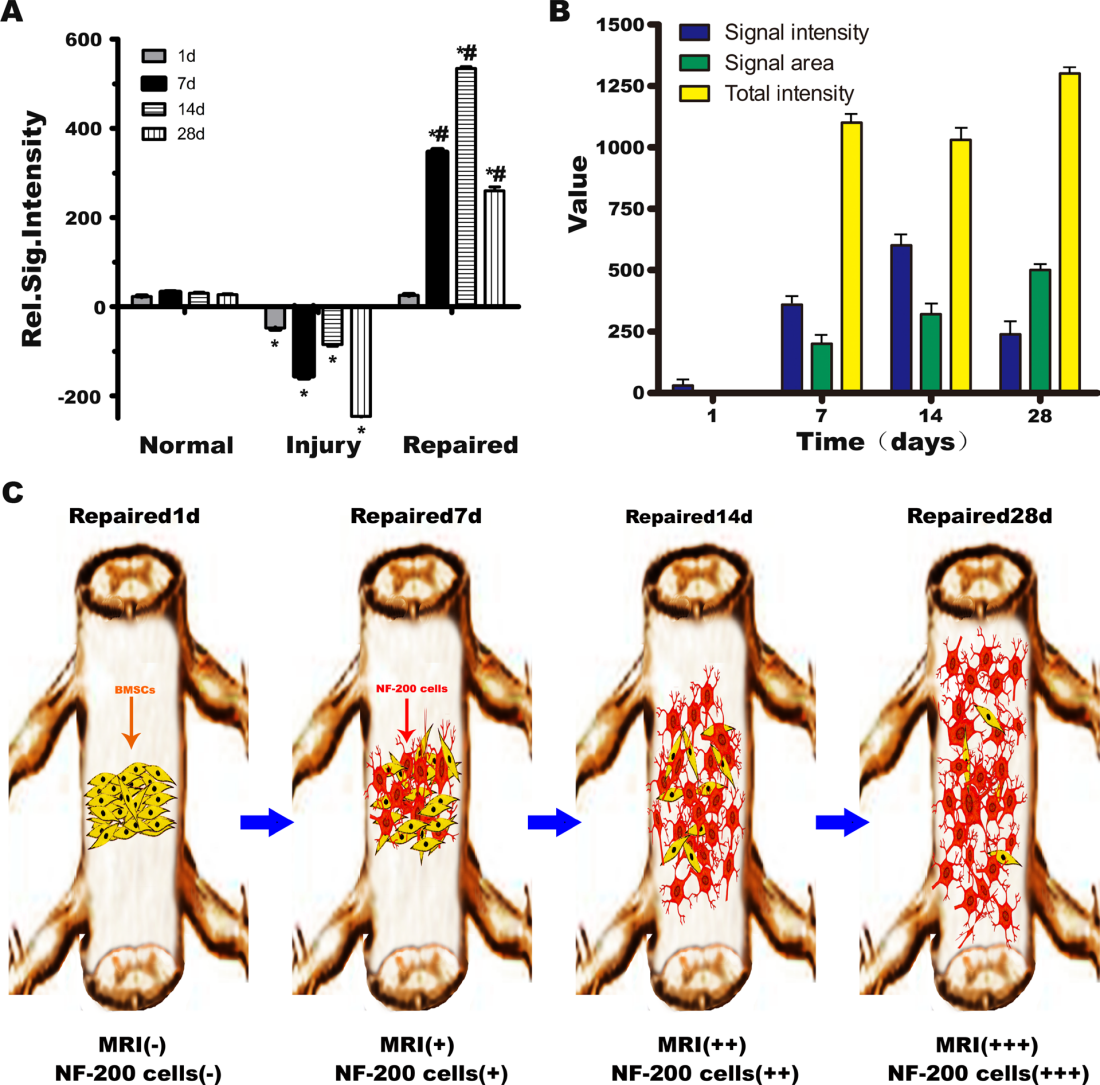
Schematic illustration of BMSCs transplantation differentiation, migration and distribution diagram (**A**) Normal group, Injury group and Repaired group rats MRI signal intensity relatively statistical histogram. *P < 0.05 verus Normal, ^#^*P* < 0.05 verus Injury. (**B**) The T1WI signal intensity distribution in Repaired group in different time (1, 7, 14, 28 days). The T1WI signal strength increased with the extension of time, at 14th day peaked, and then began to decline. The area of high signal intensity increased with the extension of time, and the biggest area of high signal intensity appeared at 28th day. The weighted total signal strength was on the rise. (**C**) Schematic illustration of BMSCs transplantation differentiation, migration and distribution diagram. In the 7th day, NF-200 positive cells were observed near the injection point, suggesting that the BMSCs had differentiated into neurons and were mainly concentrated at the site of SCI. The number of NF-200 positive cells reached a maximum at 14th day, and then gradually distributed along the segmental injury to 28th day, NF-200 positive cells distributed to both ends of the injury of spinal cord.

High T1WI signals were observed after BMSCs transplantation. In the first 7 days, transplanted cells were observed near the injection point, suggesting that the BMSCs differentiated into neurons and were mainly concentrated at the site of SCI. The cells were gathered *in situ*, where the concentration is higher, with no obvious migration. The number of cells reached a maximum at day 14 and then gradually distributed along the segmental injury. At 28 days, MRI showed high signal on T1WI, and cells continued to express NF-200, with a distribution extending to both ends of the injured cord. T1WI signal intensity and the way of measurement on T1WI signal intensity illustrateed the differentiation, migration, and distribution of BMSCs after transplantation. Red arrow indicated segmental spinal cord injury (Figure 6). What's more, after transplanted with BMSCs only labeled Gd-DTPA-FA, the normal spinal cord structure was gradually returned from 1st to 28th day in MRI imaging. However, there is no signal can be observed for T1W1 in 1st day or even until 28th day without NF-200 promoter transfection (Supplementary Figure 4).

## DISCUSSION

BMSCs are a type of adult stem cells that can differentiate into neuron-like cells *in vivo* and *in vitro* [18–20]. Transplantation of BMSCs has been accomplished *in situ* (e.g. through intravenous, intrathecal or subarachnoid injection). In the present work, we used the microsyringe to direct inject BMSCs into the SCI lesion sites, which is also minimally invasive and favored for cellular delivery into the cerebrospinal fluid. BMSCs have been observed to migrate and accumulate in the damaged areas of the nerve tissue and promote repair in previous studies [21, 22]. In our previous studies, we transplanted BMSCs to the injury site in the spinal cord of rats and monkeys. We have observed that the transplanted cells not only survive and replicate at the site of injury but also induce a modest degree of functional recovery [5, 23].

There is currently much interest in the fate of transplanted BMSCs with regard to differentiation, migration and distribution. Most of the current understanding of the migration and distribution of BMSCs in the body has been informed by histology from dead animals based at select time points. Typical histological methods provide an imperfect picture and are unable to assess the migration and distribution of transplanted cells *in vivo* and in real-time. By tagging stem cells with magnetic markers in the current study, we demonstrated the possibility of continuous and dynamic tracking of transplanted cells *in vivo* and *in vitro* using MRI. Using software for three-dimensional reconstruction [24], we are able to assess the location of cells at specific time points and also trace the movement of the cells through space. Magnetic tracer technology using magnetic markers in the body is already widely used for the stem cell transplantation treatments of brain, heart, liver and kidney damage [25]. It has also been used in the *in situ* stem cell transplantation treatment of SCI [26]. In comparison to the traditional pathology methods, magnetic tracer technology offers multiple practical advantages. Magnetic markers are mostly superparamagnetic markers, such as SPIO. These types of molecules are widely used to trace stem cells transplanted to heart, brain, and kidney [27]. In the SCI model, bleeding caused by *in-situ* injection of stem cells is often difficult to avoid [28], and the presence of blood causes reduction in T2 signal intensity, thereby influencing experimental results. Unlike the superparamagnetic markers, Gd-based markers have a high signal intensity on T1-weighted images, so it is free from the T2 interference caused by bleeding and other factors. In theory, it may be more suitable for SCI *in situ* tracer stem cell transplantation, but the use of Gd-tags is limited by diminished resolution. Thus, research efforts have been underway synthesize analogues of these Gd derivatives, but they have not yet become readily-used due to considerations about safety [29].

To perform our experiments, we adopted efficient new nano materials to mark stem cells, and constructed water-insoluble Gd markers using an enzyme activation strategy. The original Gd markers were converted into water-soluble Gd markers that could be activated by the cleavage of the ester bond. Water-insoluble Gd markers were transferred into BMSCs and at the same time cells were infected with lentiviral vectors containing the NF-200 promoter and the lipase gene. The NF-200 promoter is located upstream of the lipase gene, such that, when the BMSCs differentiated into neuron-like cells expressing NF-200, the vector would induce the secretion of lipase into cytoplasm. In the cytoplasm, lipase hydrolyzes the lipid shell and releases the water-soluble Gd markers resulting in a high signal on T1WI [30]. This allows for highly selective imaging. If none of the stem cells differentiate into NF-200 positive neuron-like cells, the transcription of the lipase gene would not be induced, and no signal would be observed for T1WI. Similarly, a previous study reported that transplanted superparamagnetic iron oxide-labeled BMSCs can be tracked by MRI in the SCI lesion, but only observed on T2W1 signals and the functional follow-up was just limited to 4 days because of the cell division and particle degradation [31]. In contrast, the new nano materials used in our recent work can be more selectively imaging and stably tracking during the SCI recovery, which will be helpful in the long-term dynamic functional assessment.

Louie [11] constructed a type of Gd markers packaged in galactose, which was not found to block water exchange with the surrounding tissue and could not be used with MRI. Additionally, when the galactose enzyme gene was introduced into cells, it could produce galactose to decompose the aliphatic chain, and “take off the shell”. While Gd could be normally visualized by MIR, if cells were not transfected with the galactose gene they could not be visualized. Himmelreich [32] observed dendritic cells (DC) *in vivo* using a similar strategy. The researchers added a lipid “shell” to Gd-DTPA, and the shell could be decomposed by lipase produced by dendritic cells. The Gd-DTPA was transferred into precursor cells of dendritic cells, and the cells were transplanted into mouse brains. If the cells differentiated into dendritic cells, they would secrete lipase and release Gd-DTPA from its lipid “shell”, allowing visualization. The Himmelreich study pioneered the strategy of using enzyme-activated reporters to visualize the dynamic differentiation and function of transplanted cells in the body.

High T1WI signals were observed after BMSCs transplantation. In the first 7 days, transplanted cells were observed near the injection point, suggesting that the BMSCs differentiated into neurons and were mainly concentrated at the site of SCI. The cells were gathered *in situ*, where the concentration is higher, with no obvious migration. The MRI signal intensity reached a maximum at day 14 and then gradually distributed along the segmental injury. At 28 days, MRI showed high signal on T1WI, and cells continued to express NF-200, with a distribution extending to both ends of the injured cord. Figure 6 illustrates the differentiation, migration, and distribution of BMSCs after transplantation. Imaging with MRI at different time points can help provide clinical insight into the repair of SCI by BMSCs.

It is difficult to use MRI to image the rat spinal cord due to the high threshold of detection for T1, the small size of the animal, and the anatomy of the injury site [33]. We optimized the scan sequence in order to obtain satisfactory images of the spinal cord of living rats. As seen histologically, the increased connectivity of spinal cord neural fibers in the experimental group is morphologic evidence that the transplanted BMSCs differentiate into neuron-like cells. Through the organization free water molecules, diffusion motion imaging allowed comparison of the organization and structure of nerve fibers as well as directional information [31]. Diffusion tensor imaging of the bundle (coursing together tensortractography, DTT) allowed for an objective assessment of the function and connection of the spinal cord. However, there were certain physiologic aspects that impaired DTI (e.g. breathing, heart and aorta beating, and motion artifacts caused by esophageal peristalsis).

DTT demonstrated that the spinal cord fibers were still interrupted from day 1 to day 28 in the Injury group. In contrast, the Repaired group of, new nerve fibers were visible as early as day 7. At 28 days, DTT (Figure 4) showed that some new fibers had partly connected to the normal spinal cord. Thus, DTI appears to have reflected the actual circumstances of spinal cord repair. These experiments provide further support that DTI is a non-invasive and non-radioactive method that can be used for other animal experiments and for the detection and diagnosis of clinical neurological diseases.

We rarely observed functional recovery and nerve reconnection in the Injury group rats. In those rare cases, the scaffold formed by the gelatin sponge may be responsible for the slight recovery [34]. Baligand [35] applied absorbable gelatin sponges as biological scaffolds to study transplanted neural stem cells and treat SCI. The material not only filled the gap of SCI but also provided three-dimensional space for axonal growth and mechanical support. The use of gelatin sponge allowed for controlled release of BMSCs to the area of local damage and may have buffered against any negative impact that other methods of transplantation would induce in the local microenvironment. In general, the absorbable gelatin sponge acts as a good biological scaffold that degrades in the body, and the adhesive gelatin surface allows axons to extend through the damage area and reduce the glial scar. The sponge appears to have a beneficial effect on cell survival and helps avoid spinal cord contracture and the formation of a cavity.

Interestingly, the gelatin sponge demonstrated a hemostatic effect, reducing local blood loss after SCI. While there was no gross evidence of bleeding, local damage was monitored by CT examination. High T1WI signals were identified as bleeding caused by the introduction of transplanted cells or the Gd tag. The use of CT to observe spinal cord usually necessitates the injection of contrast agents to display the spinal cord lesions. Acute hemorrhage on CT showed as high density in our experiments, with no need for injection of contrast. In cases where the MRI also show a high signal on T1WI, Gd signaling may mark the development of the transplanted cells. One day after injury, we did not observe an obvious T1WI signal, but we did see a high density using CT that indicated the presence of hemorrhage. To identify cells tagged by Gd, we looked for signals that were negative upon CT examination but positive on MRI T1WI.

There was no additional risk of using CT to identify hemorrhage since, to analyze the CT images, digital methods were used to strengthen the contrast and amplify any differences in color. After post-processing, the structural changes and local tissue damage could be observed by the naked eye. It was also possible to observe the differentiation of transplanted cells and the structural reshaping in post-processing images. The early soft tissue within the color was observed to be shaded and fuzzy with a swollen character, and this appearance may be associated with cellular differentiation (Supplementary Figure 1).

Several features of the Gd-labeled lipase associated nanomaterial were helpful in assessing T1WI signal changes that may reflect differentiation of BMSCs into neurons. The dynamic imaging process was enabled by the activation of lipase expression, which accurately demonstrated the distribution and migration of transplanted cells. Using this method, we were able to track the specificity of transplanted cells *in vivo*. However, previous study shown that the lipase-mediated solubilisation of the nanomaterial leads to the formation of a bisamide derivative of the DTPA ligand, which is well-known to be poorly stable, especially in the intracellular compartments [36], thus might introducing serious risks of long-term toxicity. Therefore, the concern is of importance for our further study. MRI T2WI changes were also observed in the spinal cord of experimental subjects (paritcularly in visualizing the formation of holes). However, our MRI T2-weighted images were somewhat obscured by the likely presence of scar tissue associated with newborn nerve fibers and their close proximity to regenerating spinal cord tissue. Within the Injury group, the T2-weighted signal suggested that the nerve fibers might have been reconnected on day 28. BBB scores also confirmed spinal cord repair. DTT and the biopsy results proved the function of DTI MRI imaging of spinal cord repair nerve fibers connecting accurately.

In summary, we synthesized a high efficiency, low toxicity, and enzyme activated MR molecular probe that could be used as a noninvasive tracer in live animal models. When transplanted at the site of SCI, BMSCs were found to dynamically differentiate, migrate, and distribute themselves among the nervous tissue. The MRI results confirmed that BMSCs transplanted into the injured area migrate directionally to survive. MRI was able to not only demonstrate neuronal differentiation of transplanted cells but also allowed dynamically observation of BMSC migration *in vivo* (Cover Figure 1). Due to its high resolution, non-invasive nature, and the lack of ionizing radiation, MRI should be considered as an ideal method for tracing the migration and distribution of stem cells in the body. Our experimental results may serve as a reference for future clinical applications in the transplantation of BMSCs.

**Cover Figure 1:**
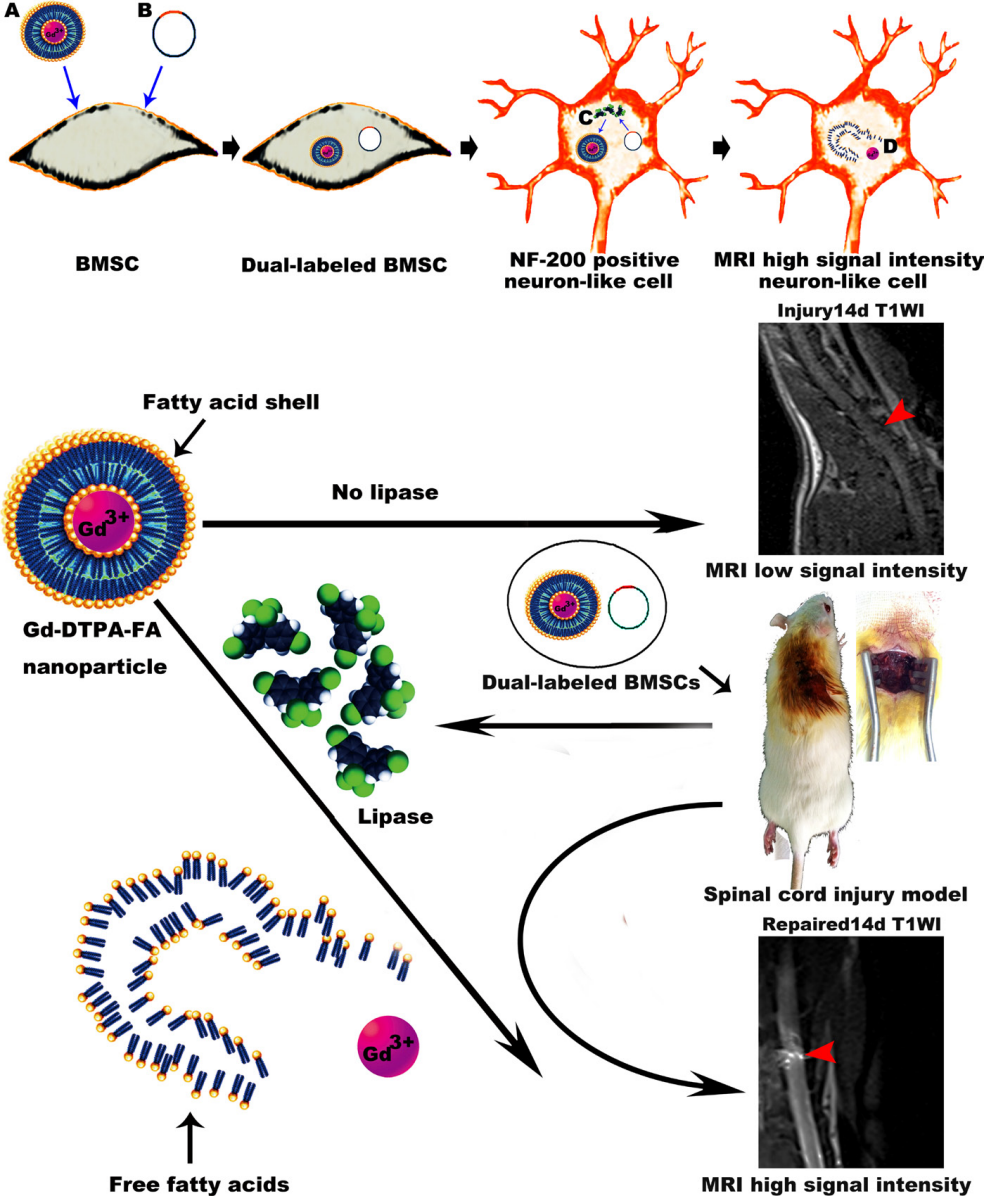
Schematic illustration for the paper's design

## MATERIALS AND METHODS

### Ethical statement

All methods mentioned in this section were carried out in accordance with the approved procedures. All animal experiments protocols were performed following the ethical guidelines and approved by the National Animal Care and Use Committee of Sun Yat-sen University, Guangzhou, China.

### Materials

Hoechst 33342 was purchased from CST (Beverly, MA, USA). Anti-rat lipase antibody was purchased from Abcam (Cambridge, UK). Dulbecco's modified Eagle's medium (DMEM) was purchased from GIBCO (Invitrogen, USA). Adult Sprague-Dawley (SD) rats (weighing 250～300 g) were purchased from the animal experiment center of Sun Yat-sen University. The endothelial lipase lentiviral vector plasmids containing the NF-200 promoter were purchased from the Shanghai Ji Kai chemical technology co., LTD. Absorbable gelatin sponge was purchased from the sigma (USA). All other chemicals were purchased from Sinopharm Chemical Reagent (China) unless otherwise noted.

### Extraction and multi-directional differentiation of BMSCs

BMSCs were extracted as previously described [27]. The expression of specific surface antigens of BMSCs were identified by flow cytometric analysis. The multi-directional differentiation of BMSCs was examined in three separate ways. First, BMSCs were induced to undergo adipogenesis and osteogenesis as previously described [37]. BMSCs were also induced into neurocytogenesis in the following manner. Selected BMSCs (P4 generation) were inoculated into cell culture dishes with 10-cell climbing plates at a concentration of 1 × 10^5^ cells per ml. After cells demonstrated confluency, the neurocytogenesis inducer was added to the media. As follows: 1 mM/ml β-mercaptoethanol into DMEM/F12 (including 10%FBS) culture medium was added to each well and incubated for 24 h. The culture medium was removed and replaced along with the addition of 2 mM/ml β-mercaptoethanol, 10% induction agent and 20 μM/ml TCS2210, and cells were allowed to incubate for 48h. Every 24 hours for 3 weeks, the culture media was replaced in each well with half the concentration of inducer (from 100% to 50%). Cellular immune fluorescence detection of P4 BMSCs induced into neuron-like cells was performed after the 3 week periodusing the Mouse Anti-NF-200 (1:100), Mouse Anti-Nestin (1:100), Rabbit Anti-NSE (1:5000), Rabbit Anti-NeuN (1:100) antibodies. Immunofluorescence analyses of cells, with Hoechst 33342 stained nuclei in blue, was performed using laser confocal microscopy sample of BMSCs induced neuron-like cells expressed by NF-200, NSE.

### Establishment of a spinal cord transection injury model

A total of 45 male SD rats weighting 250～300 g were randomly divided into 9 groups, with 5 rats in each groups (designated as Normal group, Injury group, 1 d group, 7 d group, 14 d group, 28 d group, 1 d repaired group, 7 d Repaired group, 14 d Repaired group, and 28 d Repaired group;). All the rats, with the exception of the Normal group, were anesthetized using 10% chloral hydrate (3.5 mg/kg). The vertebral plates were then excised to expose the T12 spinal cord. As described previously [38], we determined that the iliac crests, which can be located by palpation through the intact skin, are a useful landmark to locate the adjacent L6 vertebra and spinous process, which lie immediately between the iliac crests. Then, the last rib is palpated and its medial aspect is at the junction with the T13 vertebra. Counting the spinous processes sequentially rostrally from L6 to T12 was then used to confirm the location of the T12 vertebra. Transection of the spinal cord at the T12 level was performed using a taper scalpel to make a 2 mm transection injury split at spinal cord. Succesful performance of the operation result in paralaysis of the the lower limbs (BBB score = 0). For postoperative care, penicillin was injected into the peritoneum once a day (5 mg/kg/d) to prevent infection, for 1 week. Additionally, the urinary bladder was manually stimulated twice daily to assist urination until rats could urinate automatically. The animal survival rate in our present work was about 90%.

### Transplantation procedure of BMSCs

After the allotted experimental times, rat spinal cords were transected and transplanted with an absorbable gelatin sponge (Sigma, USA). The preparation of the nanomaterial was according to our previous work [15]. Briefly, the Gd-DTPA-FA particles are spheroidal with the size between 40–60 nm, and the amount of Gd payload is about 10^–5^ M. For cell labeling, cells (5 × 10^5^) were incubated together with 20 μl Gd-DTPA-FA (MW 1057 kD) complexes for 6 h under standard cell culture conditions (CO_2_ incubator: 37°C, 5% CO_2_). Rats in the Repaired groups received Gd-DTPA-FA-BMSCs (10 μl), which was injected by microsyringe, and rats in the Injury group received injection of PBS. The Gd-DTPA-FA and NF-200 promoter double labeled BMSCs were transplanted at an approximate density of 100,000 cells per microliter and with a Gd concentration of 0.5 M. The wound was closed after the transplantation by suturing overlying muscle and skin.

### CT scan and post-processing

High resolution CT scans of the spinal cord were performed using a 64-section multi-detector row CT scanner (Toshiba-aqullion, Japan) with the following parameters: Tube voltage: 120 KV; Current intensity: 200 mAs; Rebuild volume: 0.5 mm; Rebuild spinal tube in coronary and sagittal view: 0.1 mm; Soft tissue calculate and bone calculate. Post-processing was performed using a Toshiba post-processing work station to adjust image sharpness and contrast as well as to analyze a selected humorous curve to magnify low density contrast and select pseudo-color blending. Each animal was placed at the center of the CT scanner and underwent unenhanced scout scanning through the pancreas for selection of the appropriate transverse level for the CT study. Coronal and sagittal views of the area of injury on the spinal cord were processed into 3D graph and combined with 2D coronal and sagittal views as a reference. CT graph post-processing curve parameters, such as transparency, hue, saturation and gray level curve, are listed in the Supplementary material (Supplementary Figure 3).

### MRI imaging and post-processing

Before MR imaging and contrast agent administration, all animals were anesthetized with an intraperitoneal injection of chloral hydrate (3.5 mg/100 g body weight) and kept under anesthesia for the duration of the experiment. MR images were acquired using a 3.0-T MRI scanner (MAGNETOM Verio, A Tim system, Siemens, Munich, Germany). Rats were placed in the center of an 8 pathway small-animal special coil (mouse coil), diameter 5 cm, length 7.5 cm, along the vertical direction and placed on the scanning bed. All rats received coronal and sagittal T1WI (T1-weighted image) and T2WI (T2-weighted image) scans. 3D-T1W1 and DTI sequence scan, and DTT post-processing were measured by Siemens Verio 3D neuro software. Scan parameters were as follows:

T1WI scan parameters: Turbo SE; Repetition time: 580 msec; Echo time: 12 msec; Echo chain Length: 2; Flip angle: 140°; Field of view: 66 mm × 85 mm; Acquisition matrix: 200 × 256; Interpolation: on; Reconstruction matrix: 400 × 512; Slice thickness: 0.6 mm; Slice distance: 0.06 mm; Slices: 9; Bandwidth: 300 Hz/px; Average: 4; Total time: 3 m 11 s; The plane spatial resolution: 0.16 mm × 0.16 mm; SAT1.

T2WI scan parameters: Turbo SE; Repetition time: 2500 msec; Echo time: 88 msec; Echo chain Length: 12; Flip angle: 120°; iPAT; Field of view: 66 mm × 85 mm; Acquisition matrix: 200 × 256; Alice thickness: 0.6 mm; Alice distance: 0.06 mm; Alices: 9; Bandwidth: 350 Hz/px; Average: 5; Total time: 3 m 31 s; The plane spatial resolution: 0.33 mm × 0.33 mm; SAT1.

3D-T1WI scan parameters: MPRAGE-3d; Repetition time: 1750 msec; Echo time: 4.53 msec; Inversion time: 900 msec; Echo chain Length: 1; Flip angle: 9°; SENSE, iPAT = 2; Field of view: 50 mm × 65 mm; Acquisition matrix: 200 × 256; Slice thickness: 0.25 mm; Slice distance: 0.00 mm; Slices: 80; Bandwidth: 150 Hz/px; Average: 3; Total time: 8 m 41 s; The plane spatial resolution: 0.25 mm × 0.25 mm; Iso. SAT1.

DTI scan parameters: EP; Repetition time: 6900 msec; Echo time: 97 msec; Echo chain Length: 1; Flip angle: 90°; SENSE, iPAT = 2; Field of view: 50 mm × 55 mm; Acquisition matrix: 86 × 86; Interpolation: on; Reconstruction matrix: 172 × 172; Slice thickness: 1 mm; Slice distance: 0.1 mm; Slices: 35; Bandwidth: 1002 Hz/px; EPI = 128; Orientation = 20; Average: 1; Total time: 3 m 54 s; The plane spatial resolution: 0.29 mm × 0. 32 mm: FS; SAT1.

The T1WI signal intensity was measured by taking the average of the transplant centers with the most signal, and proximal signals were separated by the average of the two normal spinal vertebrae at the former, the latter was the incremental reduction of high signal intensity. The imaging data was used to generate normalized histograms of relative signal intensity. MRI signal intensity was used for univariate analysis of variance.

### Perfusion and paraffin section

Animals were anesthetized with 10% chloral hydrate, and animals were perfused by intra-aortic administration of 500ml 4% paraformaldehyde. The T10～L2 spinal cords were put in 4% paraformaldehyde for post-fixation and preserved by refrigeration at 4°C before paraffin embedding. A microtome was used to section 8 μm score-cut and cross-cut specimens. All specimens were cryopreserved at –20°C.

### Histologic analysis and immunofluorescent analysis

Sections of the T10～L2 spinal cord specimens of all groups were stained with Nissl`s stain and observed under light microscope. The medullar sheath for each sample was analyzed by electron microscopy. Briefly, medullar tissue was fixed by glutaric dialdehyde and osmium tetroxide and then embedded in epoxy resin. The number of posterior funiculus myelinated axons were counted in 8 separate fields (at 1000× magnification) for each specimen, in triplicate. Microscopic fluorescence images of the spinal cord tissues were obtained from experimental animals using an upright fluorescence microscope (Olympus, BX51, Tokyo, Japan). The tissue sections were stained with Mouse Anti-NF-200 (1:100) antibody overnight. After washing three times with PBS, the slides were stained with Alexa Fluor^®^ 647-AffiniPure Goat Anti-Mouse IgG (Jackson ImmunoResearch, USA) (1:100) for 45 min. Finally, the sections were counterstained with Hoechst33342 and analyzed using ImagePro Plus 6.0 software (Media Cybernetics, Bethesda, MD, USA).

### Locomotor function assessment

Rats in the Normal, Injury and Repaired groups were evaluated for movement function according the BBB (Beattie, Bresnahan Locomotor Rating Scale) grading standard [38]. BBB function scores were recorded for rats at different time points (1~8 weeks after establishment of Normal, Injury, and Repaired groups).

### Statistical analysis

Statistical analysis was performed using Graph Pad Prism 5.0 (La Jolla, CA, USA). All data were analyzed to determine mean±SD. Error bars denote the SD in all the results. Statistical analysis of data was performed using Student's *t*-test with one-way analysis of variance (ANOVA). Differences between groups were considered statistically significant at the level of *P* < 0.05. All statistical tests were performed with SPSS software (version 17.0, SPSS Inc., IBM, Chicago, IL, USA).

## SUPPLEMENTARY MATERIALS FIGURES



## Abbreviations

SCI: Spinal cord injury
BMSCs: Bone marrow mesenchymal stem cells
MRI: Magnetic resonance imaging
NF-200: neurofilament-200
DTI: Diffusion Tensor Imaging
DTT: Diffusion Tensor Tractography
BBB: Basso Beattie and Bresnahan Locomotor Rating Scale
