# Supplementary material for: Magnetic resonance imaging tracking and assessing repair function of the bone marrow mesenchymal stem cells transplantation in a rat model of spinal cord injury
Source: Oncotarget. 2017 Aug 1;8(35):58985–99. doi: 10.18632/oncotarget.19775 (PMC5601708; doi:10.18632/oncotarget.19775)
Supplement: Supplementary file 1 [file oncotarget-08-58985-s001.pdf]

Magnetic resonance imaging tracking and assessing repair function of the bone marrow mesenchymal stem cells transplantation in a rat model of spinal cord injury

SUPPLEMENTARY MATERIALS

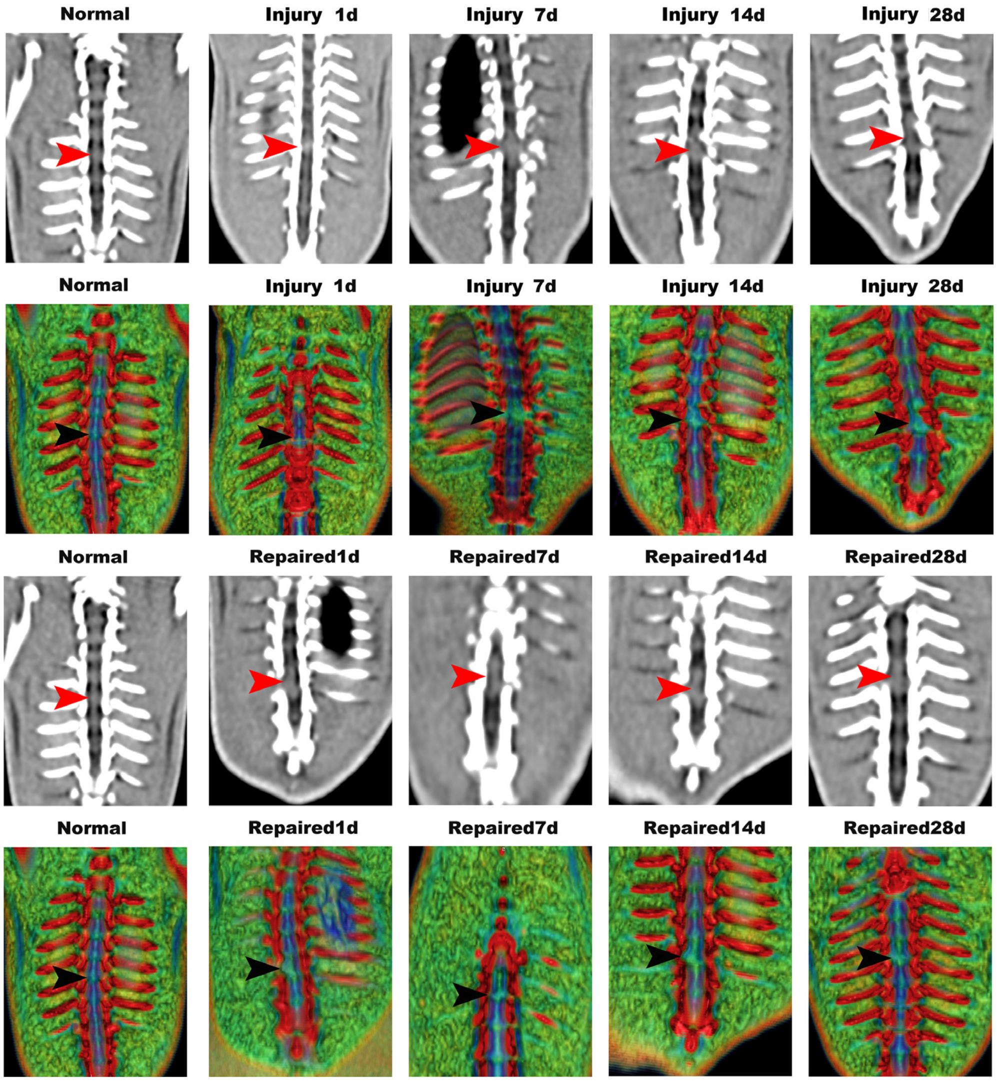

Supplementary Figure 1: CT images and post-processing coronary CT. In Normal group, the rat spinal cord was continuous and uninterrupted. Arrows indicated the segmental SCI. The hemorrhages occurred in spinal cord in CT images both Injury group and Repaired group at 1st day, and then hemorrhages disappeared in the following days. In post-processing color images, the boundary between injuried spinal cord and adjacent tissue was distinct. The green hematoma was different from blue spinal cord in Injury group. Blue thick funiculose tissue connected to the normal blue spinal cord in Repaired group.

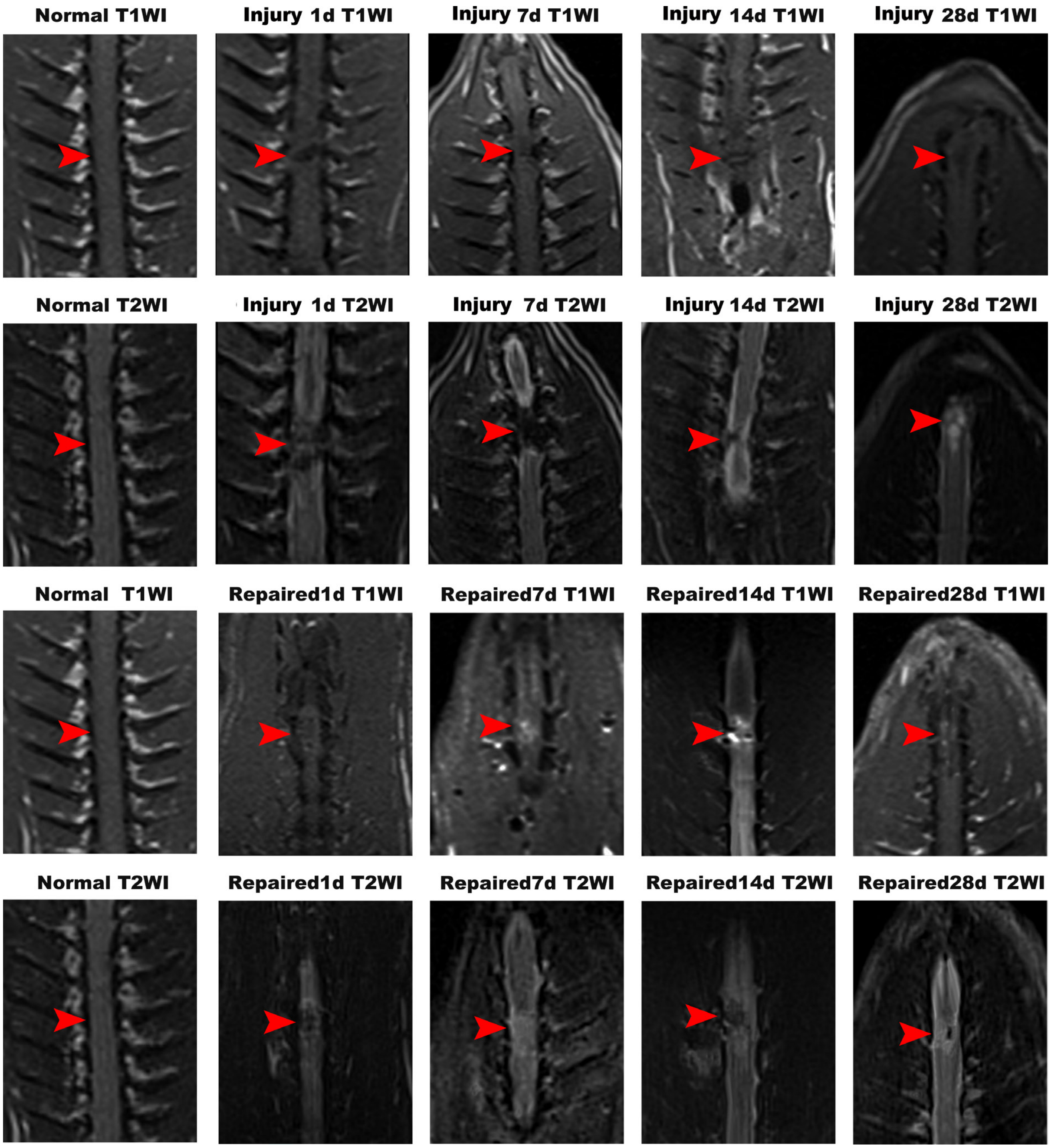

Supplementary Figure 2: T1WI and T2WI coronary MRI imaging. Normal group, Inury group and Repaired group in the rat SCI model at 1 d, 7 d, 14 d and 28 d. Arrows indicated the segmental SCI.

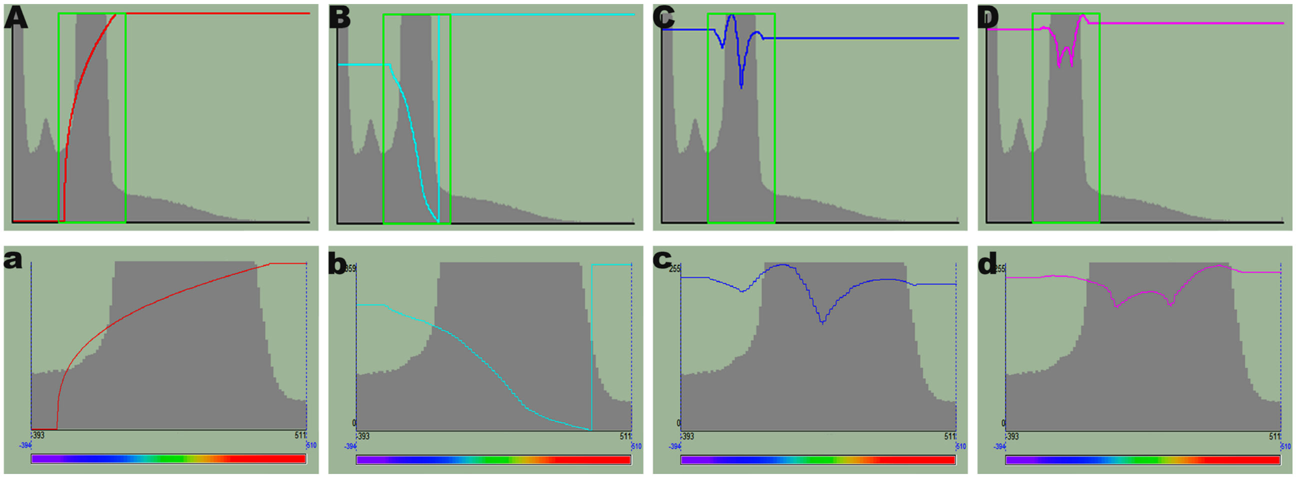

Supplementary Figure 3: CT image post-processing curve. (**A**) transparency curve; (**B**) hue curve; (**C**) the saturation curve; (**D**) grey value curve; a, b, c, d for the corresponding local amplification figure.

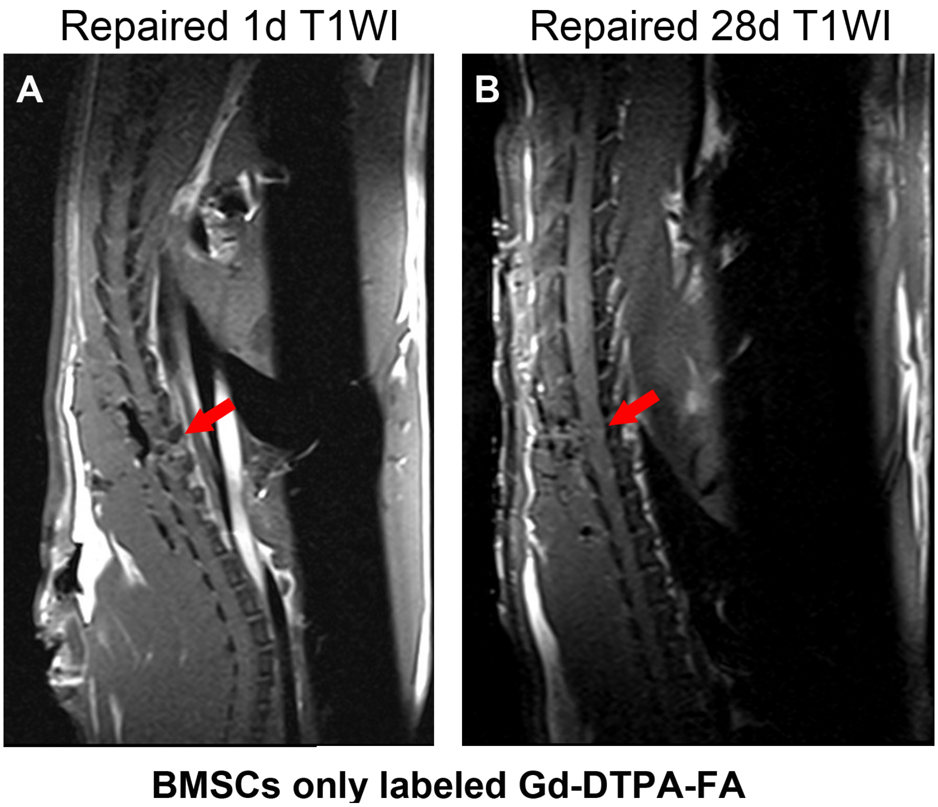

Supplementary Figure 4: MRI imaging show no signal for T1WI after transplanted with BMSCs only labeled Gd-DTPA-FA. (**A**) Repaired group in the rat SCI model at 1 d. (**B**) Repaired group in the rat SCI model at 28 d. Arrows indicated the segmental SCI.
